# A Turn-On Quinazolinone-Based Fluorescence Probe for Selective Detection of Carbon Monoxide

**DOI:** 10.3390/molecules28093654

**Published:** 2023-04-22

**Authors:** Akari Tange, Naoya Kishikawa, Yusuke Sakamoto, Mahmoud El-Maghrabey, Mitsuhiro Wada, Naotaka Kuroda

**Affiliations:** 1 Graduate School of Biomedical Sciences, Course of Pharmaceutical Sciences, Nagasaki University, 1-14 Bunkyo-machi, Nagasaki 852-8521, Japandr_m_hamed@mans.edu.eg (M.E.-M.); 2 School of Pharmaceutical Science, Nagasaki University, 1-14 Bunkyo-machi, Nagasaki 852-8521, Japan; 3 Department of Pharmaceutical Analytical Chemistry, Faculty of Pharmacy, Mansoura University, Mansoura 35516, Egypt; 4 Faculty of Pharmaceutical Sciences, Sanyo-Onoda City University, 1-1-1 Daigakudori, Yamaguchi 756-0884, Japan; m-wada@rs.socu.ac.jp

**Keywords:** carbon monoxide, quinazolinone, fluorescence probe, test paper, metal free

## Abstract

Carbon monoxide (CO) is a toxic, hazardous gas that has a colorless and odorless nature. On the other hand, CO possesses some physiological roles as a signaling molecule that regulates neurotransmitters in addition to its hazardous effects. Because of the dual nature of CO, there is a need to develop a sensitive, selective, and rapid method for its detection. Herein, we designed and synthesized a turn-on fluorescence probe, 2-(2′-nitrophenyl)-4(3*H*)-quinazolinone (NPQ), for the detection of CO. NPQ provided a turn-on fluorescence response to CO and the fluorescence intensity at 500 nm was increased with increasing the concentration of CO. This fluorescence enhancement could be attributed to the conversion of the nitro group of NPQ to an amino group by the reducing ability of CO. The fluorescence assay for CO using NPQ as a reagent was confirmed to have a good linear relationship in the range of 1.0 to 50 µM with an excellent correlation coefficient (r) of 0.997 and good sensitivity down to a limit of detection at 0.73 µM (20 ppb) defined as mean blank+3SD. Finally, we successfully applied NPQ to the preparation of a test paper that can detect CO generated from charcoal combustion.

## 1. Introduction

Carbon monoxide (CO) is a gas produced by the incomplete combustion of organic compounds and is ubiquitously found in the smoke from heating appliances and automobile exhaust. CO can bind more strongly to the hemoglobin in red blood cells than oxygen; thus, the blood cannot carry oxygen, resulting in hypoxia [1,2]. In addition to preventing oxygen delivery, CO can bind to many other hemoproteins such as myoglobin, Cytochrome P450, and mitochondrial cytochrome oxidase to alter these functions. CO poisoning is usually caused by inhalation of excess CO and is the most common form of fatal air poisoning [3,4]. The symptoms of CO poisoning are headache, nausea, dizziness, weakness, vomiting, chest pain, and confusion. For example, fatal poisoning from CO generated by charcoal combustion often occurs. CO is a colorless and odorless gas that is difficult to perceive and can easily cause poisoning accidents [5,6,7]. On the other hand, CO is produced endogenously in vivo through heme degradation by heme oxygenase. CO has been reported to be concerned with a series of physiological processes such as vasodilation, anti-inflammatory response, and neurotransmission responses as important cell signaling molecules and is attracting attention as the third most bioactive molecule after H_2_S and NO [8,9,10]. In addition, it has been reported that abnormal blood CO concentrations are observed in patients with Alzheimer’s disease and cardiovascular diseases [8]. Against this background, it is necessary to develop analytical methods for monitoring CO in air and in vivo.

Although numerous approaches have been established to detect CO, such as gas chromatography [9,10], Fourier-transform infrared spectroscopy (FT-IR) [11], and electrochemical analysis [12], these methods have drawbacks such as the complexity of the equipment and the difficulty with real-time on-site detection. In contrast, fluorescence-based assays are simple, rapid, and suitable for real-time on-site monitoring [13,14,15,16,17]. Since He’s group [18] and Chang’s group [19] reported a probe for CO detection in 2012, various fluorescent probes have been developed to detect CO [20,21,22,23,24,25,26]. Wang et al. have developed a selective fluorescence cell imaging probe for CO. This fluorescent probe was composed of a genetically encoded dimeric heme protein (CooA) for CO sensing and yellow fluorescent protein variants. CO causes displacement-induced conformational changes in CooA, which lead to yellow fluorescence enhancement from the yellow fluorescent proteins. The probe showed acceptable selectivity towards nitric oxide, molecular oxygen, and cyanides [18]. However, the synthesis of this probe is laborious and time-consuming. Feng’s group successfully monitored CO in vivo based on the Tsuji–Trost reaction in the presence of Pd [27]. Moreover, Chang’s groups have developed a cyclopalladated derivative of boron dipyrromethene difluoride (BODIPY) where the palladium quenches the fluorescence of the BODIPY owing to the known heavy-atom electronic effect of palladium. Upon addition of CORM-3 as a source of CO, it leads to a release of palladium in its reduced metallic form Pd(0) and forms a fluorescence carbonylated BODIPY [19]. However, these probes have drawbacks such as the addition of palladium, a heavy metal of potential toxicity, the requirement for heating at high temperatures, and long reaction times that reach one hour. On the other hand, Zhang et al. developed a ratiometric fluorescent probe for CO based on hemocyanin moiety [20]. Additionally, a palladium-free CO fluorescent probe was developed based on coumarin moiety; however, this probe synthesis is somewhat complicated, and the Stokes shift is short (about 45 nm) [22].

**Figure 1:**
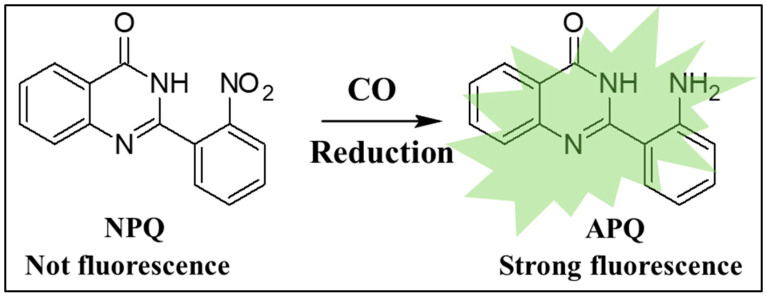
Fluorogenic sensing of CO through its reduction action on NPQ forming APQ.

The detection of toxic gases is of immense significance. Hence, more attention has shifted to the development of a highly sensitive, simple, cost-effective, and rapid sensor for their detection [17]. Our research group has been focusing on developing new fluorescence sensors for toxic volatile compounds and gases [28,29,30,31]. Hence, we aim to develop a new fluorescence turn-on probe for CO with long Stokes shift and good selectivity and sensitivity. Quinazolinone derivatives possess excellent photophysical properties. Their fluorescence relies on the turn-on of internal charge transfer (ICT), which makes them possess long Stokes shifts. Additionally, quinazolinone derivatives are characterized by their aggregation-induced emission (AIE) [32,33]. Owing to their excellent luminescence properties, in the present study, we developed a quinazolinone-based turn-on fluorescence probe, 2-(2′-nitrophenyl)-4(3*H*)-quinazolinone (NPQ), for the detection of CO. NPQ is itself non-fluorescent but rapidly reacts with CO and is converted to 2-(2′-aminophenyl)-4(3*H*)-quinazolinone (APQ), which emits strong green fluorescence (Figure 1). This fluorescence enhancement could be attributed to the reduction in the nitro group of NPQ to an amino group upon reaction with CO, which emits fluorescence due to the ICT process. In this work, a sensitive and selective fluorescence assay for CO with NPQ utilizing this fluorescence enhancement was developed. The reduction reaction of CO with quinazolinone was found to be rapid and is about 90% completed in 10 min, which is faster than other reported reduction-based probes for CO sensing [34]. Quinazolinone derivatives are known to have excellent fluorescence in solutions depending on the solvents due to the characteristics of their electronic spectra, which have attracted great interest due to good photophysical properties such as intense luminescence, excellent Stokes shift, and photostability [35,36,37]. In addition, since quinazolinone fluorophore exhibits AIE properties [38,39], NPQ in the solid state could be applied to the detection of CO. Therefore, we attempted to develop a test paper method to detect gaseous CO using filter paper adsorbed with NPQ.

## 2. Results and Discussion

### 2.1. Structural and Property Changes in NPQ in Reaction to CO

**Figure 2:**
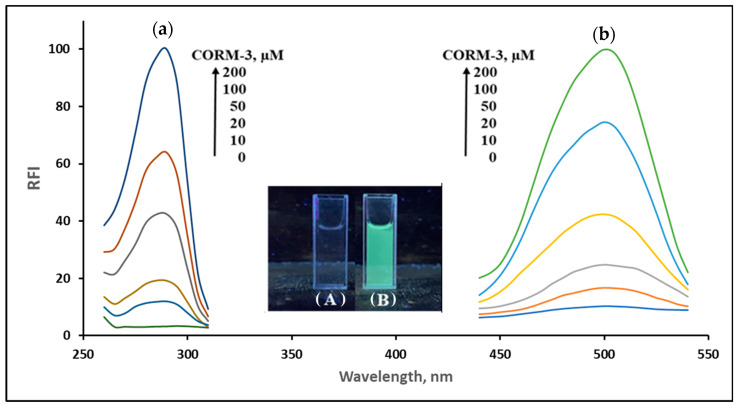
(**a**) The excitation spectra (λ_ex_ = 500 nm) and (**b**) emission spectra (λ_ex_ = 280 nm) of NPQ (30 µM) upon the addition of different concentrations of CORM-3 (0, 10, 20, 50, 100, 200 µM). The reaction time was 30 min at room temperature. The solvents for NPQ and CORM-3 are as described in the experimental section. The insets are (**A**) a photograph of NPQ (30 µM) and (**B**) a photograph of NPQ (30 µM) upon the addition of CORM-3 (100 µM). RFI refers to relative fluorescence intensity.

**Figure 3:**
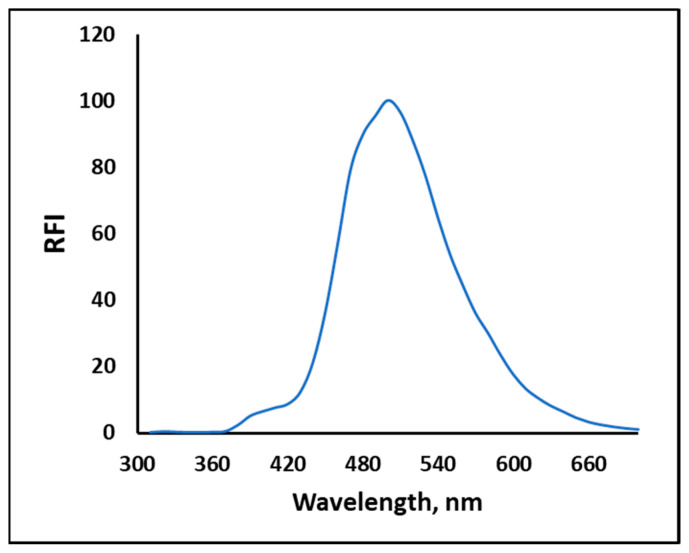
The fluorescence emission spectra of APQ (30 µM); λ_ex_ = 280 nm. The solvent for APQ was PBS buffer (pH 7.0, 10 mM, containing 30% DMSO).

In the current study, CORM-3 was used as a standard for carbon monoxide, owing to its well-known releasing capability of CO in vitro and even in vivo [40]. To examine the changes in spectral characteristics of NPQ, the excitation and emission spectra of NPQ after the reaction with CO released from CORM-3 were measured. The excitation bands from 280 nm to 290 nm and the fluorescence emission around 500 nm were increased with increasing the concentration of CORM-3 (Figure 2). As shown in the inset in Figure 2, when the NPQ solution alone was UV irradiated (solution A), no fluorescence was observed, while strong green fluorescence was observed upon the addition of CORM-3 to NPQ (solution B). These results indicate that NPQ can be used for CO determination. The fluorescence emission spectrum of the APQ solution showed fluorescence with a maximum wavelength of around 500 nm and was identical to that of NPQ after the reaction with CO (Figure 3). Therefore, this fluorescence enhancement was suggested to be caused by the substitution of an electron-withdrawing nitro group involved in the quenching of quinazolinone fluorophore for an amino group.

**Figure 4:**
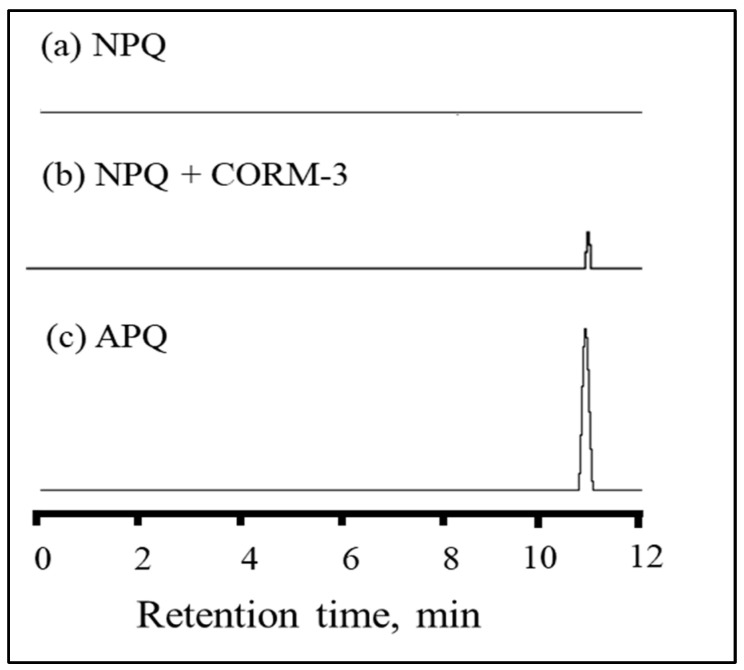
HPLC with fluorescence detection chromatograms of (**a**) NPQ, (**b**) NPQ after the reaction with CORM-3, and (**c**) APQ. HPLC conditions: column, Nacalai Cosimosil 5C18-AR-II (4.6 × 150 mm); mobile phase, CH_3_CN/H_2_O (50/50, *v*/*v*%); flow rate, 0.5 mL/min; detection wavelength, λ_ex_ = 280 nm and λ_em_ = 500 nm; injection volume, 20 µL.

To confirm the suggested reaction mechanism, HPLC experiments were performed to prove the conversion of NPQ to APQ by comparing the retention times (Figure 4). The used HPLC conditions are mentioned in Figure 4. Since NPQ is a non-fluorescent compound, a signal peak was not observed on its chromatogram (Figure 4a). On the other hand, when CORM-3 was added to NPQ, a signal peak was detected at 11 min (Figure 4b), the same retention time as that of APQ (Figure 4c). These results show that the NPQ is converted to the highly fluorescent APQ by the reducing ability of CO [21,34].

**Figure 5:**
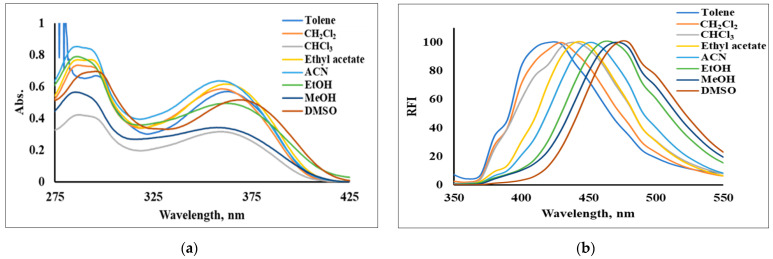
(**a**) Absorption spectra and (**b**) normalized emission spectra (λ_ex_ = 280 nm) of APQ (30 µM) in different organic solvents.

The photophysical properties of APQ in different solvents were examined to explore the fluorescence mechanism. As shown in Figure 5a, the maximum absorption wavelength of APQ hardly changed with different types of solvents; only some changes to the absorbance were observed, which could be attributed to the different physical properties of the solvents. This result indicates that the ground state of APQ did not change significantly with solvent polarity [32]. On the other hand, the maximum fluorescence wavelength of APQ was gradually red-shifted from 420 nm to 480 nm with increasing solvent polarity (Figure 5b), till it reached 500 nm upon using PBS buffer (pH 7.0, 10 mM, containing 30% DMSO) as a solvent (Figure 3). Thus, it was suggested that the formation of the ICT state upon excitation is responsible for the fluorescence emission of APQ [35,41]. The photophysical properties of APQ in different solvents are summarized in Table 1. APQ showed the highest molar absorption coefficients in acetonitrile. However, among the tested single solvents, the use of DMSO resulted in the highest quantum yield (6%) and brightness (139,200) for APQ in a single solvent. Upon using PBS buffer (pH 7.0, 10 mM, containing 30% DMSO) as a solvent (Figure 3), the quantum yield was increased five times, reaching 30%.

### 2.2. Selectivity of NPQ to CO

**Figure 6:**
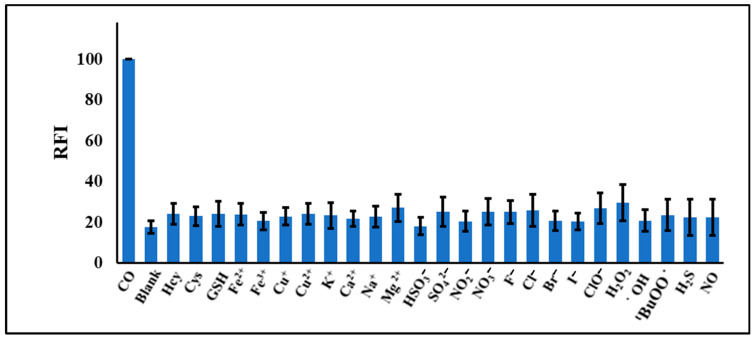
Fluorescence responses of NPQ (30 μM) at 500 nm after adding CO, reductants, reactive oxygen species, and gaseous transmitters in PBS buffer. The concentrations of CO, Fe^2+^, Fe^3+^, Cys, Hcy, Cu^+^, and Cu^2+^ were 100 µM, while the concentrations of other substances were 1 mM. The reaction time was 30 min at room temperature. The solvent for NPQ is described in the experimental section.

**Figure 7:**
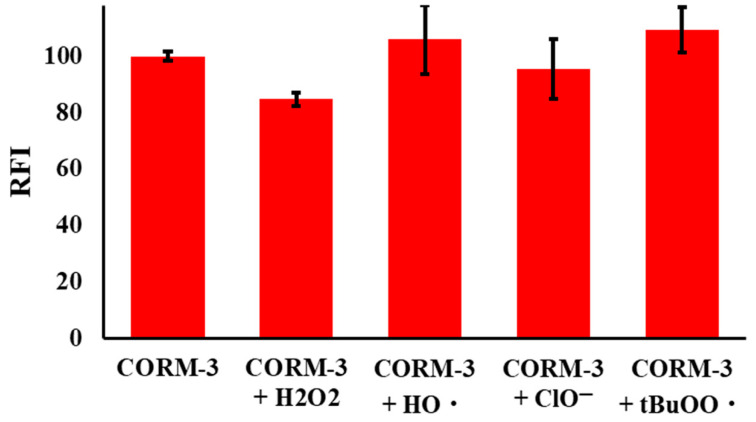
Fluorescence responses of NPQ (30 µM) upon addition of CORM-3 (100 µM) in the presence of reactive oxygen species (1 mM) in PBS buffer. The reaction time was 30 min at room temperature. The solvent for NPQ is described in the experimental section.

To evaluate the selectivity of the reaction of NPQ with CO, the change in fluorescence was measured with the addition of typical reductants, including Fe^2+^, Cys, Hcy, and GSH, reactive oxygen species, including H_2_O_2_, ^•^OH, ClO^−^, and ^t^BuOO^•^, and gaseous transmitters including H_2_S and NO. Hydroxyl radical (^•^OH) and *tert*-butoxy radical (^t^BuOO^•^) were generated by a reaction of 100 µM Fe^2+^ with 1 mM H_2_O_2_ or 1 mM TBHP, respectively. Moreover, various metal ions and anions’ possible interference was tested, including the effect of Fe (III), Fe(II), Cu(I), Cu(II), K^+^, Ca^2+^, Na^+^, Mg^2+^, sulfate, hydrogen sulfate, nitrate, nitrite, fluoride, bromide, chloride, and perchlorate. As shown in Figure 6, the fluorescence of NPQ was enhanced only when CORM-3 was added, and little change in fluorescence was observed after the addition of other substances instead of CORM-3. Furthermore, the presence of reactive oxygen species in competitive experiments did not affect the fluorescence enhancement of NPQ by CO (Figure 7). Therefore, it was confirmed that NPQ has excellent selectivity for CO.

### 2.3. Optimization of Reaction Condition and Validation of CO Determination

**Figure 8:**
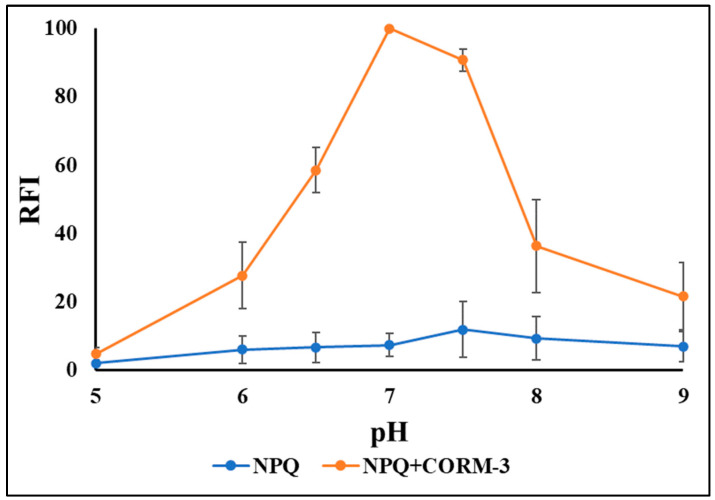
The effects of pH on the fluorescence intensity of NPQ (30 µM) upon the addition of CORM-3 (100 µM). The reaction time was 30 min at room temperature. The solvents for NPQ and CORM-3 are as described in the experimental section, except for pH.

**Figure 9:**
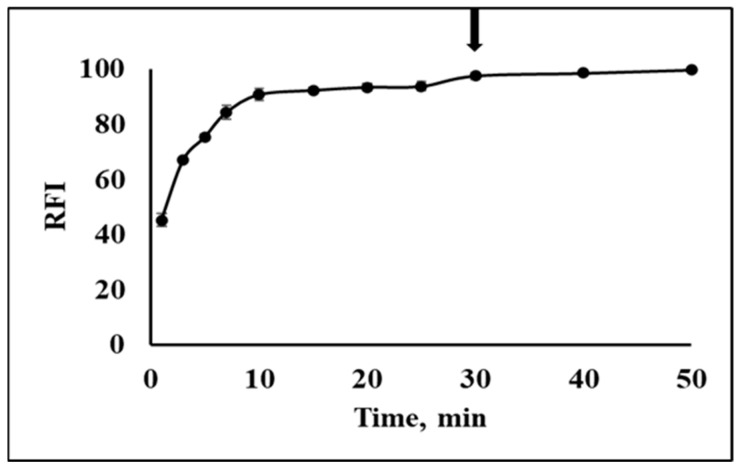
The effect of reaction time on the fluorescence intensity of NPQ (30 µM) upon the addition of CORM-3 (100 µM). The solvents for NPQ and CORM-3 are as described in the experimental section. The arrow indicates the optimum reaction time.

We utilized NPQ to develop a simple fluorescence assay for the determination of CO using a spectrofluorometer. To achieve higher sensitivity, the effect of reaction conditions on the fluorescence intensity of NPQ after the reaction with CO was investigated. At first, the effect of pH on the fluorescence intensity of NPQ (30 µM) upon the addition of CORM-3 (100 µM) was investigated over a range of pH 5.0–9.0. The reaction time was 30 min at room temperature. NPQ was dissolved in PBS buffer (10 mM, pH 7.0) containing 30% DMSO (*v*/*v*), while CORM-3 was dissolved in PBS. It was found that at pH 5, no reaction occurred; however, increasing the pH led to an increase in the fluorescence intensity till it reached the maximum at pH 7. Then, as the medium became slightly alkaline, the fluorescence intensity decreased gradually (Figure 8). Hence, pH 7.0 was selected because it gave the maximum relative fluorescence intensity (RFI). The reaction between NPQ and CO proceeded smoothly, even at room temperature. Thus, the reaction time was investigated from 1 to 50 min at room temperature. The RFI increased gradually till it reached nearly the plateau at 10 min, and then the RFI remained nearly constant for at least 40 min. As the maximum and constant fluorescence intensity was obtained at 30 min, it was selected as the reaction time (Figure 9).

**Figure 10:**
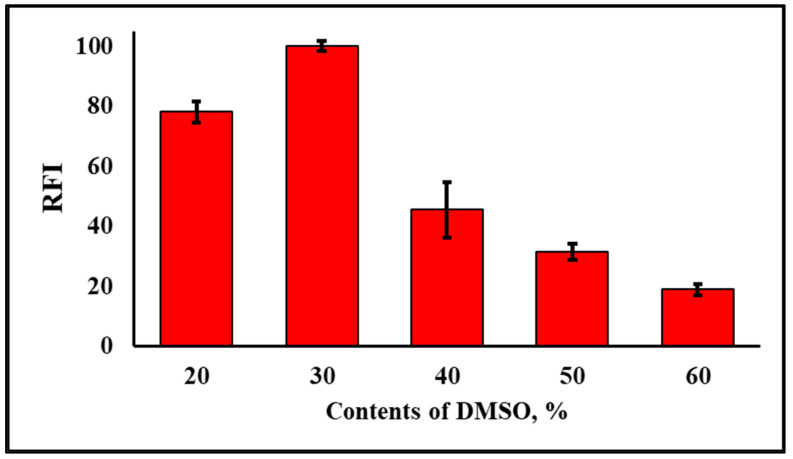
The effects of the DMSO contents in the PBS buffer (10 mM, pH 7.0) of NPQ (30 µM) upon the addition of CORM-3 (100 µM). The reaction time was 30 min at room temperature. The solvents for NPQ and CORM-3 are as described in the experimental section, except for DMSO contents.

From Table 1 and Figure 5b, increasing solvent polarity led to a red shift in the maximum fluorescence wavelength of APQ (Figure 5b), till it reached 500 nm upon using PBS buffer (pH 7.0, 10 mM, containing 30% DMSO) as a solvent (Figure 3). This demonstrates that the fluorescence emission of APQ relies on the formation of the ICT state upon excitation [35,41]. As polar solvents stabilize the charge-transfer states, the highest ICT emission is usually observed in polar solvent mixtures such as water/DMSO mixture. However, from APQ structure, it could form an intramolecular hydrogen bond, which in the presence of a high amount of water, could lead to fluorescence quenching due to the formation of mixed intramolecular and intermolecular hydrogen bonds [44]. Consequently, we investigated the contents of DMSO in the PBS buffer, and good fluorescence intensity was obtained in the 20% DMSO, which was increased with increasing DMSO % to 30. After that, increasing the DMSO amount to 40% led to a decrease in fluorescence intensity. Then, as the DMSO increased, the fluorescence intensity decreased. From the previous results, the best composition of polar solvent mixtures that stabilizes the charge-transfer states and retains the intramolecular hydrogen bond with the minimum intermolecular hydrogen bond is PBS buffer containing 30% DMSO. Hence, the best condition was using 30% DMSO, which yielded the highest RFI (Figure 10). It is noteworthy that this nonlinear relation between the solvent water content and fluorescence intensity of quinazoline derivatives is similar to what was reported by Wang et al. [33].

**Figure 11:**
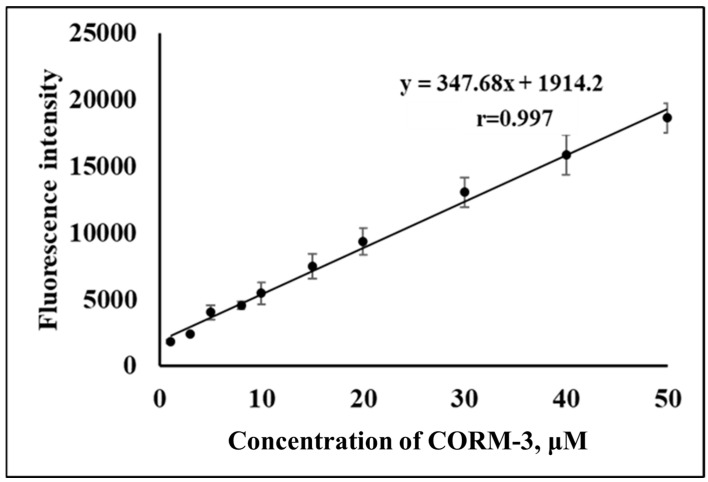
The calibration curve was constructed by plotting the concentration CORM-3 from 1.0 to 50 µM versus the fluorescence intensity.

The calibration curve was prepared under optimized conditions (Figure 11). A good linear relationship (r = 0.997) between the concentration of CORM-3 and the fluorescence intensity, measured at 500 nm after excitation at 280 nm, was obtained in the range of 1.0–50 µM. The linear regression equation was Y = 347.68X + 1914.2, where Y and X represent the fluorescence intensity and the concentration of COMR-3, respectively. The limit of detection, defined as mean blank+3SD, was 0.73 µM. The performance of the NPQ compared to previously reported probes is summarized in Table 2. Compared to another Pd-based probe that requires the addition of metal to detect CO, NPQ has comparable or relatively higher sensitivity [19,20,26,45,46,47,48]. NPQ also has the advantage of not requiring the concomitant use of the noble metal Pd. On the other hand, NPQ is superior to other reduction-based probes in that it can detect CO in a shorter reaction time than other probes and is less susceptible to interference by excitation light due to its large Stokes shift [21,34]. As can be seen in Table 2, the Stokes shift is about 220 nm, which is higher than most of the reported sensors in the literature [19,20,21,26,34,45,46,47,48]. Moreover, the sensitivity of NPQ is either higher or similar to other reduction-based probes [21,34]. Another advantage of NPQ that is worth mentioning is its excellent selectivity towards CO (Figure 6).

The repeatability of the proposed assay by NPQ was examined using different concentrations (5, 10, and 50 µM) in the calibration range either on the same day (intra-day precision) or on different consecutive days (inter-day precision). The relative standard deviations (R.S.D) for intra-day (n = 5) assays were 3.8, 4.5, and 3.8%, respectively, and for inter-day (n = 5) assays were 9.4, 5.5, and 7.0%, respectively. Therefore, the good repeatability of the proposed assay was confirmed.

It is noteworthy that the reaction between CO and NPQ that produces APQ is not reversible, as the re-oxidation of APQ to NPQ does not occur by atmospheric oxygen or oxygen dissolved in solvents; however, it needs very strong oxidizing agents such as dimethyldioxirane and potassium iodide–tert-butyl hydroperoxide [49,50]. Hence, the reversibility of our probe reaction with CO is not likely to happen, which is similar to previously reported nitro probes for CO [21,34].

### 2.4. NPQ Test Paper for Visual Detection of CO

**Figure 12:**
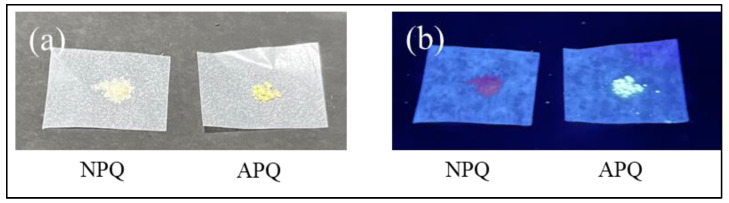
Photographs of NPQ in the solid state under (**a**) natural light and (**b**) UV irradiation with a handy UV lamp (λ_ex_ = 365 nm).

**Figure 13:**
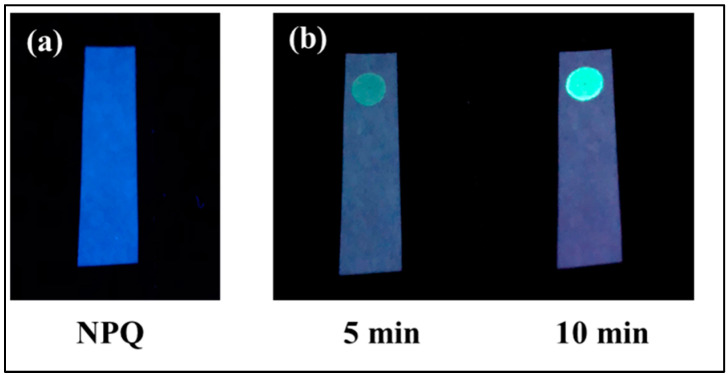
Fluorescence of the NPQ (30 µM) test paper dropped with CORM-3 solution (100 µM): (**a**) before dropping the CORM-3 solution; (**b**) 5 min and 10 min after dropping the CORM-3 solution.

Because of the AIE properties of quinazolinone fluorophores [32,33], the fluorescence of APQ can be observed in the solid state (Figure 12). Using this property, a test paper was prepared for the visual detection of CO in the vapor phase. The test paper was prepared simply by soaking a piece of filter paper with the NPQ solution and then air drying it. As shown in Figure 13, when 100 µM CORM-3 solution was dropped on the NPQ test paper, and fluorescence was observed under UV irradiation at 365 nm, fluorescence was observed at the point where CORM-3 was dropped after 5 min, and clear green fluorescence could be detected visually after 10 min.

**Figure 14:**
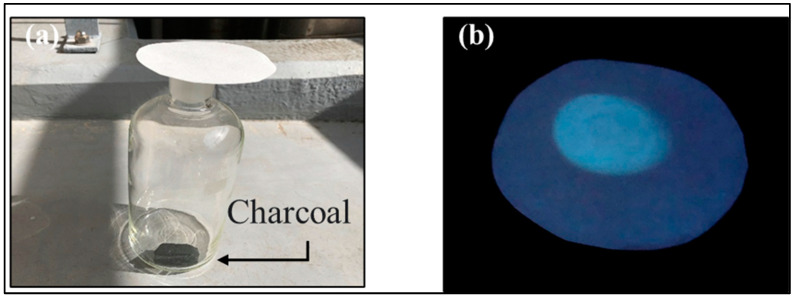
Fluorescence of the NPQ test paper exposed to smoke generated from charcoal combustion. (**a**) Photograph of test paper as it is exposed to smoke. (**b**) Fluorescence in areas exposed to smoke for 30 min.

Finally, the prepared test paper was applied to detect CO generated from the combustion of charcoal. As a result, fluorescence was observed in the area on the test paper exposed to charcoal smoke, suggesting that the CO in the smoke came into contact with NPQ adsorbed on the test paper, resulting in the fluorescence turn-on of the test paper (Figure 14). In previous reports, most fluorescent probes have used tricarbonyl dichloro ruthenium (II) dimer (CORM-2) or CORM-3 as CO-releasing agents and have not been applied to the detection of CO gas produced by combustion of organic materials [21,51,52]. On the other hand, the proposed NPQ was able to detect CO directly in charcoal smoke; the NPQ test paper has the potential for applications such as being useful for on-site monitoring of CO to alert acute CO poisoning.

## 3. Experimental

### 3.1. Material and Instruments

Purified water was obtained using Auto still WG 203 (Yamato Scientific Co., Ltd., Tokyo, Japan). *o*-Nitorobenzaldehyde, iodine, tin (II) chloride dihydrate (SnCl_2_·2H_2_O), sodium dihydrogen phosphate dihydrate, sodium hydrogen phosphate, hydrogen peroxide (H_2_O_2_), iron (II) chloride tetrahydrate, iron (III) chloride hexahydrate, copper (I) chloride, potassium chloride, calcium chloride, sodium hydrogen sulfite, sodium nitrite, sodium nitrate, and sodium sulfide nonahydrate were purchased from Wako Pure Chemicals (Osaka, Japan). Tricarbonylchloro(glycinato)ruthenium (carbon monoxide releasing molecule 3, CORM-3), sodium bromide, and *tert*-butyl hydroperoxide (TBHP) were purchased from Sigma-Aldrich (St. Louis, MO, USA). Sodium thiosulfate pentahydrate, dimethyl sulfoxide (DMSO), homocysteine (Hcy), sodium chloride, sodium sulfate, sodium iodide, and sodium hypochlorite were obtained from Nacalai Tesque (Kyoto, Japan). Cysteine (Cys) and magnesium chloride anhydrous were purchased from Kishida (Osaka, Japan). Glutathione (GSH) was purchased from Tokyo Chemical Industries (Tokyo, Japan). 1-Hydroxy-2-oxo-3-(*N*-methyl-3-aminopropyl)-3-methyl-1-triazene (NOC7, a NO donor) was obtained from Dojindo (Kumamoto, Japan). All reagents and chemicals were purchased from manufacturers and applied directly without further purification. The websites of the supporting companies for the used chemicals are listed in Table S1 (Supplementary Materials).

The fluorescence and UV–vis spectra were recorded on an RF-1500 spectrofluorometer (Shimadzu, Kyoto, Japan) and UV-1800 spectrophotometer (Shimadzu, Kyoto, Japan), respectively. The structures of NPQ and APQ were analyzed by EI-MS (*m/z*) using JMS-700N (JEOL, Tokyo, Japan), elemental analysis using Perkin Elmer 2400 Ⅱ (MA, USA), melting point measurement using ATM-02 (AS ONE, Osaka, Japan), and ^1^H NMR using Varian5-inova500 (500 Hz) spectrometer (Varian, CA, USA).

### 3.2. Synthesis of NPQ

NPQ was synthesized by a one-step reaction (Figure S1) as follows. *o*-nitrobenzaldehyde (300 mg, 2 mmol) and *o*-aminobenzamide (400 mg, 3 mmol) were dissolved in ethanol (20 mL), and then iodine (750 mg, 3 mmol) was added, and the reaction mixture was refluxed to 80 ℃ for 6 h. After cooling to room temperature, 5% sodium thiosulfate aqueous solution (200 mL) was added and allowed to stand for a few minutes. The precipitate was collected by filtration and then washed with ethanol, toluene, and then water. The residual solid was purified by silica gel column chromatography to acquire a pale-yellow solid (yield; 51.3% and m.p. 230–231 °C). The structure was confirmed by EI-MS (*m*/*z*), H^1^ NMR, and elemental analysis. The EI-MS spectrum (Figure S2) shows a molecular ion peak at 267 corresponding to [M]^+^. The ^1^H NMR spectra (Figure S3, and 500 MHz, DMSO-d_6_) δ (ppm) results were as follows: δ =7.56 (t, *J* = 7.6Hz, 1H), 7.64 (d, *J* = 8.1Hz, 1H), 7.80–7.92 (m, 4H), 8.18 (q, *J* = 7.4 Hz, 2H), 12.82 (s, 1H). The elemental analysis was calculated for C_14_H_9_N_3_O_3_; C, 62.92%; H, 3.39; N, 15.72%; found: C, 62.69%; H, 3.24%; N, 15.23%. The MS, ^1^H NMR, and elemental analysis data are very similar to those reported by Sayahiet et al., who have synthesized NPQ previously; however, they used a different synthetic approach [53]. These data and those from the previous report prove the NPQ structure with a molecular formula of C_14_H_9_N_3_O_3_.

### 3.3. Synthesis of APQ

NPQ (0.13 mmol) and SnCl_2_·2H_2_O (0.52 mmol) were dissolved in methanol (10 mL), and the reaction mixture was refluxed to 80 ℃ for 2 h. The solvent was removed by evaporation under reduced pressure. The residue was redissolved in ethyl acetate. The mixture was washed with saturated NaHCO_3_ and saturated NaCl, respectively. The resulting organic layer was dried with anhydrous MgSO_4_; then, purification was carried out by silica gel column chromatography to acquire a yellow solid (yield; 76.6% and m.p. 245–246 ℃). The structure was confirmed by EI-MS (*m*/*z*), ^1^H NMR, and elemental analysis. The EI-MS spectrum (Figure S4) shows a molecular ion peak at 237, corresponding to [M]^+^. The H^1^ NMR spectra (Figure S5 and 500 MHz, DMSO-d_6_) δ (ppm) results were as follows: δ = 6.58 (t, J = 7.5 Hz, 1H), 6.80 (d, J = 8.0 Hz, 1H), 7.05 (s, 2H), 7.18 (t, J = 7.3 Hz, 1H), 7.47 (t, J = 7.0 Hz, 1H), 7.81–7.70 (m, 3H), 8.10 (d, J = 7.8 Hz, 1H), 12.10 (s, 1H). These ^1^H NMR data are very similar to those reported by Saravanan et al. and Venkateswarlu et al., who have synthesized the APQ and its derivatives [54,55]. The elemental analysis is calculated for C_14_H_11_N_3_O; C, 70.87%; H, 4.67; N, 17.71%; found: C, 70.60%; H, 4.71%; N, 17.72%. These data and those from the previous report prove the APQ structure with a molecular formula of C_14_H_11_N_3_O.

### 3.4. General Procedure for Fluorescence Measurement of CO Released from CORM-3

To 2 mL of 30 µM NPQ in PBS buffer (10 mM, pH 7.0) containing 30% DMSO (*v*/*v*), 2 mL of CORM-3 in PBS buffer was added and mixed, and then the mixture was kept at room temperature for 30 min. Afterward, the fluorescence spectrum of the mixture was recorded at the excitation of 280 nm.

### 3.5. CO Sensing with NPQ Test Paper

NPQ test papers were prepared by immersing filter paper in an ethyl acetate solution of NPQ (30 µM) for 2 h and then air dried. After a drop of CORM-3 solution (100 µM) was added to the prepared NPQ test paper and left to stand for several minutes, fluorescence was visually observed under UV light irradiation at 365 nm. In addition, to evaluate the ability of the NPQ test paper to detect gaseous CO, charcoal was combusted in a glass jar, and the NPQ test paper was put at the opening of the jar. After the NPQ test paper was exposed to smoke generated from charcoal combustion in a glass jar for 30 min, the fluorescence of the test paper under UV light was observed.

## 4. Conclusions

In the present work, we developed NPQ, a highly sensitive and selective quinazolinone-based fluorescent probe for CO detection. NPQ could be synthesized easily in a one-step reaction using inexpensive and readily available materials. The nitro group of NPQ is reduced to an amino group by the reaction with CO to emit strong fluorescence around 500 nm. NPQ turned on its fluorescence towards CO selectively and showed very good tolerance to different interferents, including various anions and metal ions, oxidants, reductants, and even free radicals. NPQ could detect CO with good sensitivity down to the detection limit of 0.73 µM. Finally, NPQ could be applied to develop a test paper for the visual detection of CO in the vapor phase.

## Figures and Tables

**Table 1:** Summary of the photophysical properties of APQ in different solvents.

Solvent	Absorbance λ-max (nm)	Fluorescence λ-max (nm)	Stokes Shift	Molar Absorption Coefficients	PLQY (%) ^a^	Brightness ^b^
Toluene	296	420	124	22,233	0.3	6,670
CH_2_Cl_2_	287	430	143	24,467	1.3	31,807
CHCl_3_	287	440	153	14,100	3.9	54,990
Ethyl acetate	293	440	147	25,667	1.8	46,201
ACN	286	450	164	28,467	2.2	62,627
Ethanol	286	460	174	26,300	2.9	76,270
Methanol	285	470	185	18,833	2.6	48,966
DMSO	295	480	185	23,200	6	139,200
30% DMSO in PBS buffer	280	500	220	22,567	30	667,010

^a^ PLQY (photoluminescence quantum yield) calculated using quinone sulfate as a reference fluorophore adopting the procedure described by Hariharasubramanian and Ravichandran [42]. ^b^ Brightness = Extinction Coefficient (ε) × Fluorescence Quantum Yield (Φ) [43]

**Table 2:** Comparison of the proposed NPQ with the previously reported CO detection probes.

Probe Name	Detection Reaction	Wavelengths(λex/λem, nm)	Reaction Time (min)	Detection Limit (µM)	Ref.
Hcy-CO	Pd-based, Tsuji–Trost reaction	410/515 410/600	15	3.8	[20]
Flav-1	Pd-based, Tsuji–Trost reaction	411/603	15	3.19	[45]
MPVC-1	Pd-based, azido carbonylation reaction	424/550	10	100 ppm (3.57 × 10^3^ µM)	[26]
COP-1	Pd-mediated carbonylation reaction	475/507	None	1	[19]
COP-3E-Py	Pd-based, carbonylation reaction	521/535	60	None	[46]
Pd-BNP-OH	Pd-based, nanostructure probe	405/510	240	1.9	[47]
MENap-Pd	Pd-based, demetallation reaction	435/532	30	1.4	[48]
Na-CM-ER	Nitro group reduction	430/520	70	0.42	[34]
LysoFP-NO_2_	Nitro group reduction	440/530	45	0.6	[21]
NPQ	Nitro group reduction	280/500	30	0.73	This work

## Data Availability

The data will be available upon reasonable request.

## Supplementary Materials

The following supporting information can be downloaded at: https://www.mdpi.com/article/10.3390/molecules28093654/s1, Table S1: The websites of the supporting companies for the used chemicals in the study. Figure S1: Synthesis of NPQ and APQ; Figure S2: EI-MS spectrum of NPQ; Figure S3: 1H NMR spectrum of NPQ with enlargement at the interest area between 7 and 8.5 ppm as an inset; Figure S4: EI-MS spectrum of APQ; Figure S5: 1H NMR spectrum of APQ with enlargement at the interest area between 6.5 and 8.5 ppm as an inset.

## Sample Availability

Samples of the compounds NPQ and APQ are available from the authors.
