# Supplementary material for: A Turn-On Quinazolinone-Based Fluorescence Probe for Selective Detection of Carbon Monoxide
Source: Molecules. 2023 Apr 22;28(9):3654. doi: 10.3390/molecules28093654 (PMC10180483; doi:10.3390/molecules28093654)
Supplement: Supplementary file 1 [file molecules-28-03654-s001.zip › molecules-2344751-supplementary.pdf]

Supplementary Materials

A Turn-On Quinazolinone-Based Fluorescence Probe for Selective Detection of Carbon Monoxide

Table S1. The websites of the supporting companies for the used chemicals in the study

Company	Website
Yamato Scientific Co., Ltd., Tokyo, Japan	https://www.yamato-scientific.com/
Wako Pure Chemicals (Osaka, Japan)	http://www.wako-chem.co.jp/english/
Sigma-Aldrich (St. Louis, USA)	https://www.sigmaaldrich.com/JP/en
Nacalai (Kyoto, Japan)	https://www.nacalai.co.jp/global/
Tokyo Chemical Industries (Tokyo)	https://www.tcichemicals.com/JP/en/
Dojindo (Kumamoto, Japan).	https://www.dojindo.com/ASIA/

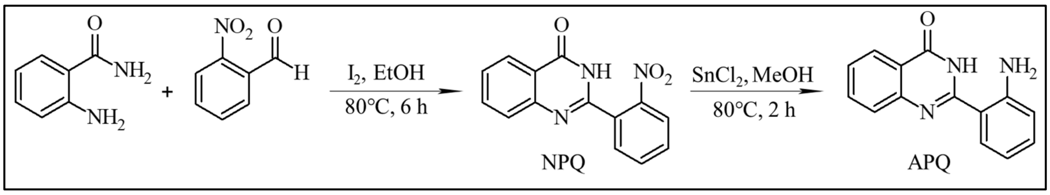

Figure S1. Synthesis of NPQ and APQ.

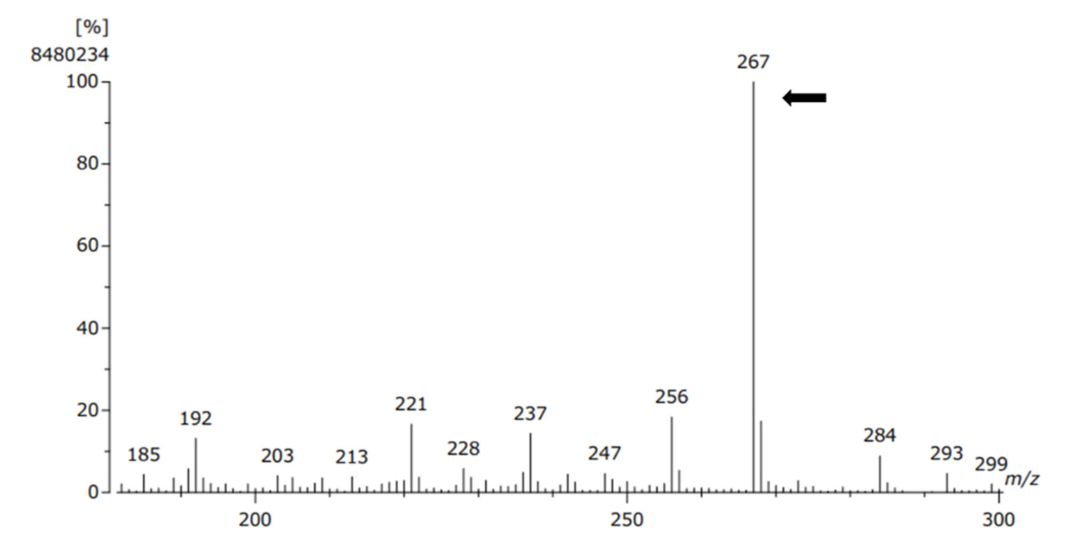

Figure S2. EI-MS spectrum of NPQ.

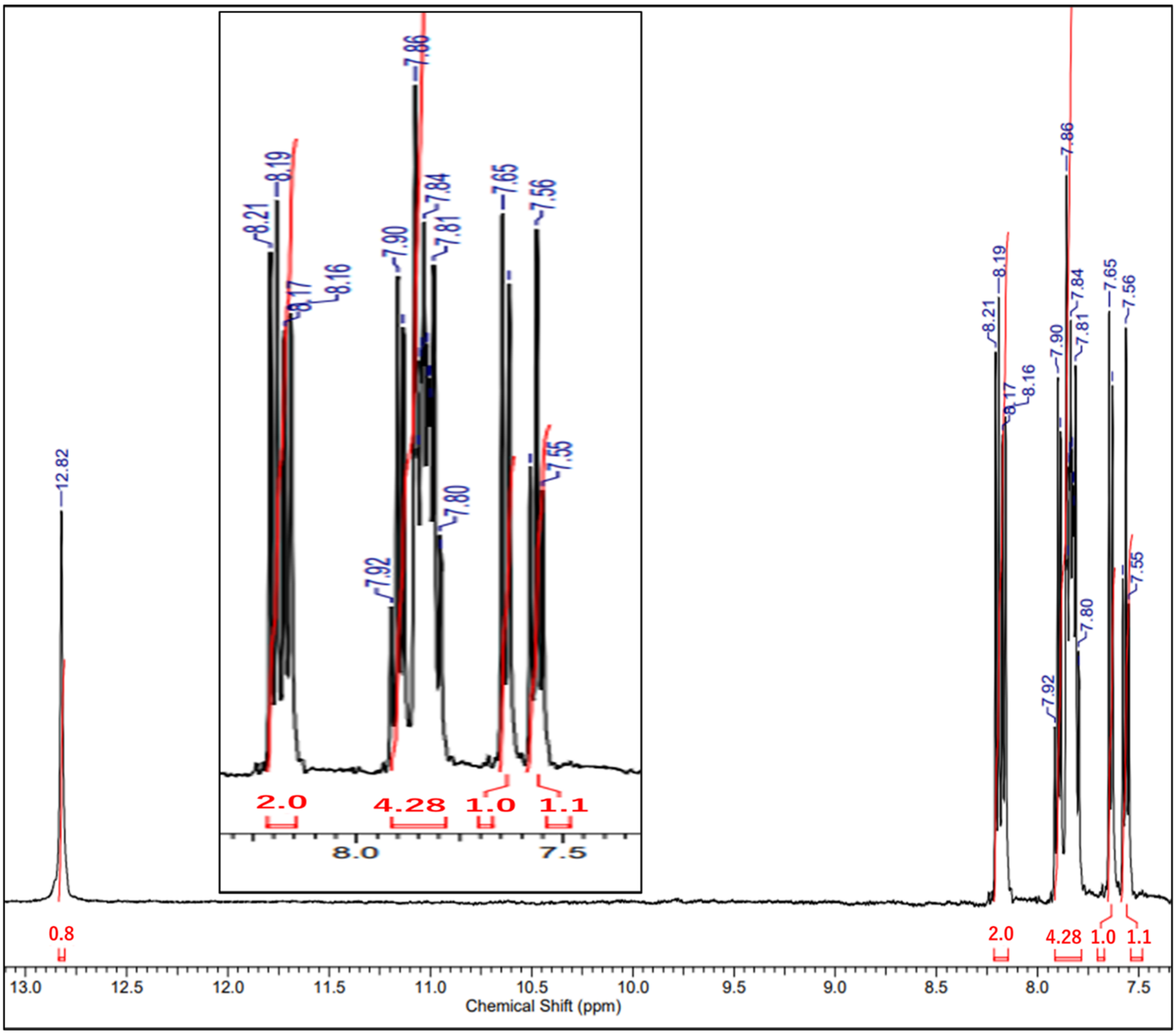

Figure S3. ^{1}H NMR spectrum of NPQ with enlargement at the interest area between 7 and 8.5 ppm as an inset.

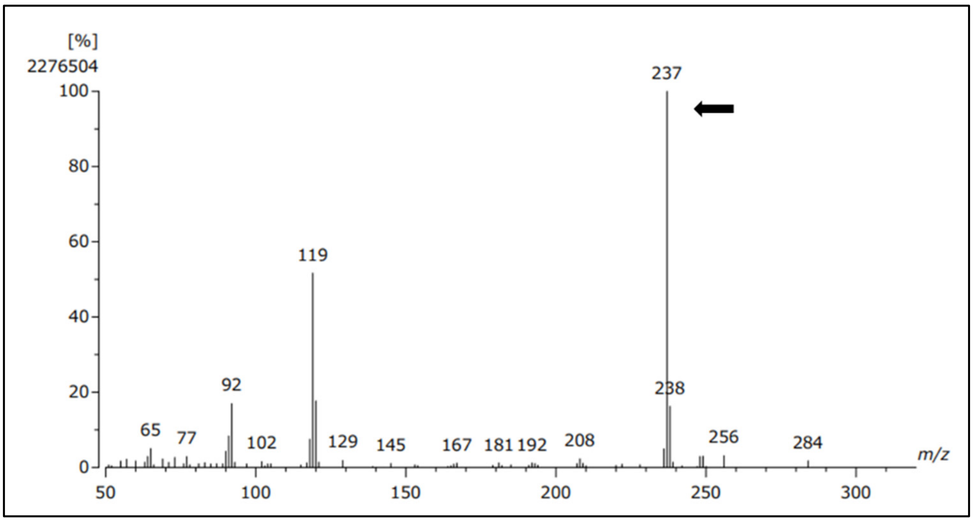

Figure S4. EI-MS spectrum of APQ.

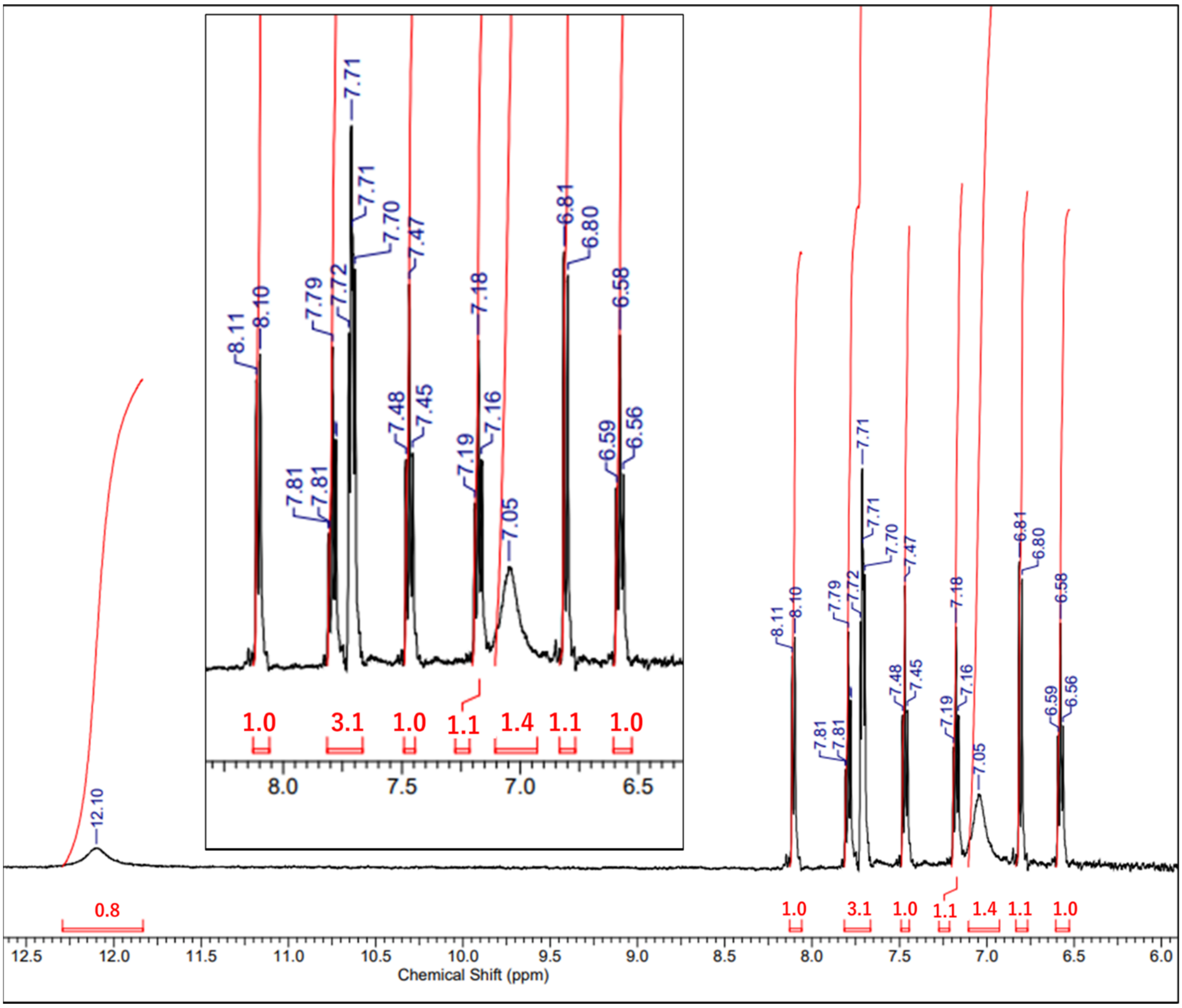

Figure S5. ^{1}H NMR spectrum of APQ with enlargement at the interest area between 6.5 and 8.5 ppm as an inset.
